# The complete mitochondrial genome of *Macrourus whitsoni* (Gadiformes, Macrouridae)

**DOI:** 10.1080/23802359.2020.1773339

**Published:** 2020-06-05

**Authors:** Seung Jae Lee, Tae-Eul Im, Euna Jo, Eunkyung Choi, Young Min Chi, Jin-Hyoung Kim, Jeong-Hoon Kim, Hyun Park

**Affiliations:** a Division of Biotechnology, College of Life Sciences and Biotechnology, Korea University, Seoul, Korea; b Unit of Research for Practical Application, Korea Polar Research Institute (KOPRI), Incheon, Korea; c Division of Polar Life Science, Korea Polar Research Institute, Incheon, Korea

**Keywords:** Mitochondria genome, PacBio, Macrouridae, *Macrourus witsoni*

## Abstract

The complete mitochondrial genome of *Macrourus witsoni* was determined in this study by the Long-read Technology, such as PacBio Sequel System. The Long-read Technology, which can sequence continuously the whole vertebrate mitochondrial genome, allows more accurate genomes to be completed. The circular form of its mitochondrial genome was 16,714bp, which contained 13 protein-coding genes, 22 tRNA, and 2 rRNA. The gene orders of *M.witsoni* was identical to that of the other species of Macrouridae family. Phylogenetic analysis indicated *M. witsoni* was mostly close to *C.kishinouyei* in the Macrouridae family.

*Macrourus whitsoni* (Regan, 1913), whose common name is Whitson’s grenadier, is distributed in the 600–1500 m deep sea of the Circumpolar (generally confined inside the Convergence except in the Falkland Islands area), characterized with two Dorsal spines, relatively large eyes, large mouths, and thin lips (Cohen et al. 1990). There are four classified species in the *Macrourus* genus, but the complete mitogenome has not been reported yet, although six complete genomes have been reported in the Macrouridae family. Their complete mitochondria genomes were used for classification through phylogenetic analysis.

The sample was collected from Ross Sea (77°05′S, 170°30′E on CCAMLR Subarea 88.1), Antarctica, and DNA was isolated using the conventional phenolchloroform method. The specimen was deposited at the Earth Biocollection in the Division of Biotechnology, Korea University, with the accession number KAN0001030. Using the covaris G-tube (Covaris, Woburn, MA, USA), we generated 20 kb fragments by shearing genomic DNA according to the manufacturer’s recommended protocol. The SMRTbell library was constructed by using SMRTbell™ Template Prep Kit 1.0 (Pacific Biosciences, Menlo Park, CA, USA). The SMRTbell library was sequenced using SMRT cells (Pacific Biosciences, Sequel™ SMRT^®^ Cell 1 M v3) using sequencing kit (Sequel Sequencing Kit 2.1) and 1 × 600 minutes movies were captured for each SMRT cell using the Sequel (Pacific Biosciences) sequencing platform. PacBio subreads for mitochondria assembly were filtered out using 16 s rRNA and COI gene sequences of the other Antarctic fishes. *De novo* assembly for mitogenome was performed by CANU assembler (Koren et al. 2017). The assembled mitogenomic sequences were retrieved into MITOS web service (Bernt et al. 2013) for mitogenomic annotation.

The complete mitogenome of *M. whitsoni* (GenBank Number: MT157320) was 16,714 bp long that encodes 37 genes, including 13 protein-coding genes, 22 transfer RNAs, and 2 ribosomal RNAs (Figure 1). The GC contents was 41.11%. The evolutionary history was inferred by using the maximum likelihood method and JTT matrix-based model (Jones et al. 1992). The tree with the highest log likelihood (–35781.95) is shown. Initial tree(s) for the heuristic search were obtained automatically by applying neighbor-join and BioNJ algorithms to a matrix of pairwise distances estimated using a JTT model, and then selecting the topology with superior log likelihood value. The tree is drawn to scale, with branch lengths measured in the number of substitutions per site. This analysis involved 19 amino acid sequences. There were total of 3732 positions in the final dataset. Evolutionary analyses were conducted in MEGA X (Kumar et al. 2018).

**Figure 1.**
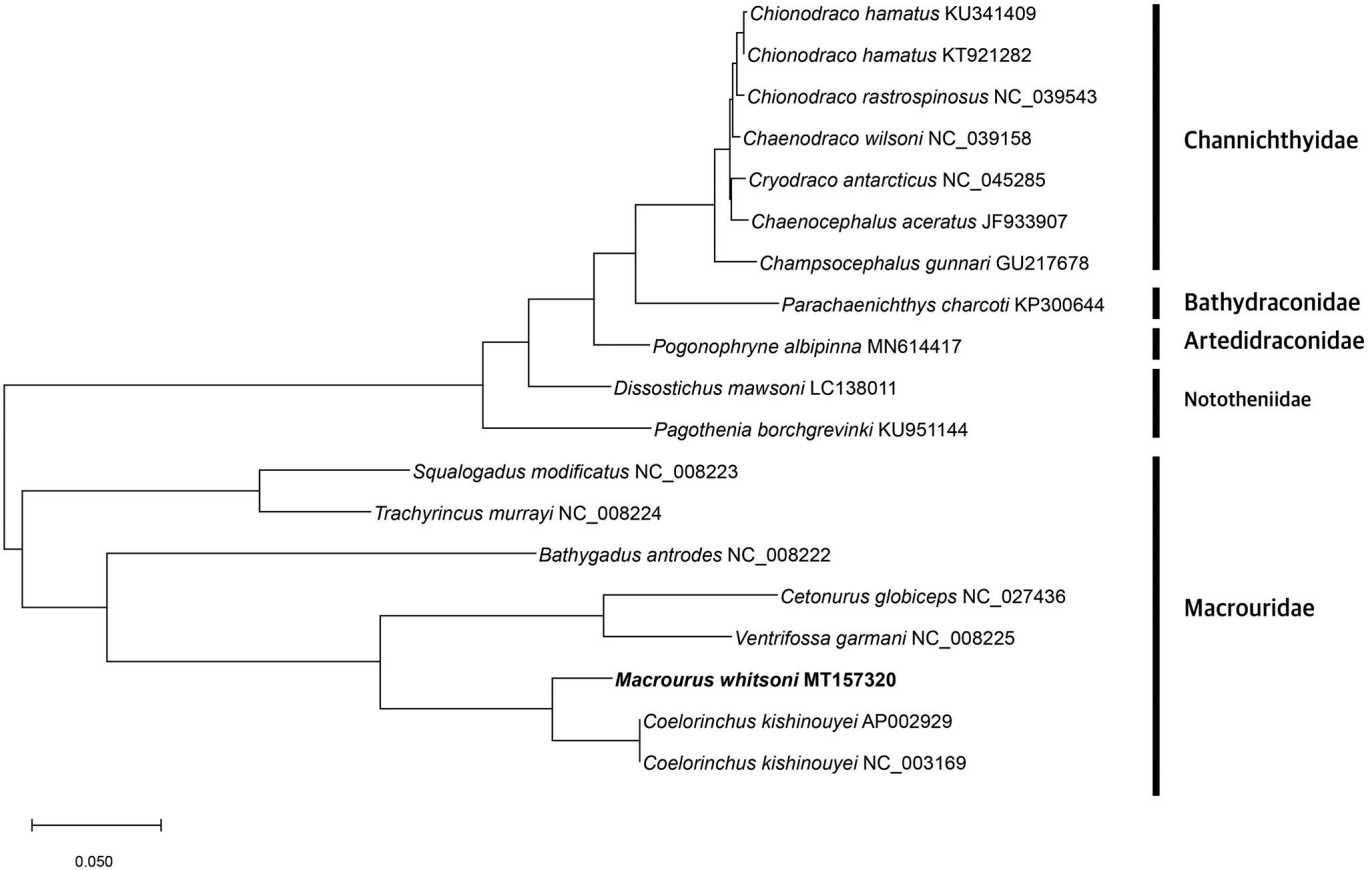
Phylogenetic tree was constructed with protein sequences of 13 protein-coding genes of 19 Antarctic fishes which were belong to Channichthyidae, Bathydraconidae, Nototheniidae, Artedidraconidae and Macrouridae families including *M. whitsoni* using MEGA X software. The maximum likelihood method and JTT matrix-based model were used. Scientific name and GenBank number are indicated for each species.

## Data Availability

The data that support the findings of this study are openly available in NCBI under the accession MT157320 (https://www.ncbi.nlm.nih.gov/nuccore/MT157320.1/).
